# Real-time submillisecond single-molecule FRET dynamics of freely diffusing molecules with liposome tethering

**DOI:** 10.1038/ncomms7992

**Published:** 2015-04-24

**Authors:** Jae-Yeol Kim, Cheolhee Kim, Nam Ki Lee

**Affiliations:** 1 Department of Physics, Pohang University of Science and Technology, Pohang 790-784, Korea; 2 School of Interdisciplinary Bioscience and Bioengineering, Pohang University of Science and Technology, Pohang 790-784, Korea

## Abstract

Single-molecule fluorescence resonance energy transfer (smFRET) is one of the powerful techniques for deciphering the dynamics of unsynchronized biomolecules. However, smFRET is limited in its temporal resolution for observing dynamics. Here, we report a novel method for observing real-time dynamics with submillisecond resolution by tethering molecules to freely diffusing 100-nm-sized liposomes. The observation time for a diffusing molecule is extended to 100 ms with a submillisecond resolution, which allows for direct analysis of the transition states from the FRET time trace using hidden Markov modelling. We measure transition rates of up to 1,500 s^–1^ between two conformers of a Holliday junction. The rapid diffusional migration of *Deinococcus radiodurans* single-stranded DNA-binding protein (SSB) on single-stranded DNA is resolved by FRET, faster than that of *Escherichia coli* SSB by an order of magnitude. Our approach is a powerful method for studying the dynamics and movements of biomolecules at submillisecond resolution.

## Abstract (web summary)

Single-molecule fluorescence resonance energy transfer is widely used to probe biomolecular dynamics, but is limited by its temporal resolution. Here, Kim *et al*. push the limit to submillisecond for the duration of tens of milliseconds by tethering target molecules to liposomes in buffer solutions.

## Graphical abstract

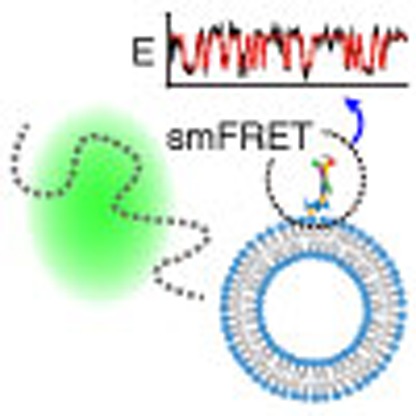

Advances in single-molecule techniques have made it possible to investigate the heterogeneity of reaction mixtures and the dynamics of unsynchronized reactions of biomolecules, such as protein/RNA folding, conformational changes, protein diffusion on DNA and enzymatic reactions of machinery proteins, to elucidate detailed molecular mechanisms^1^,^2^,^3^. Of the currently available single-molecule techniques, single-molecule fluorescence resonance energy transfer (smFRET) has been widely used to study the dynamics and heterogeneity of biomolecules because smFRET can sensitively measure distances at the level of 2–10 nm through noninvasive optical measurements^4^,^5^,^6^. Two types of measurement have been most widely used for smFRET detection. One is the use of a confocal excitation scheme to detect one molecule at a time as it freely diffuses in solution; this technique is often called diffusion-based smFRET^7^,^8^,^9^,^10^,^11^. The other technique uses a total internal reflection microscope to detect a single molecule immobilized on a glass surface (immobilized smFRET)^2^,^5^. Immobilized smFRET has been used to study the real-time dynamics of single molecules, whereas diffusion-based smFRET has been used for and is well suited to observing the molecular heterogeneity in a buffer solution^7^,^8^,^12^,^13^.

Despite its capability of observing the real-time dynamics of a single molecule for a long period of time, immobilized smFRET is limited in its temporal resolution^5^. The temporal resolution of immobilized smFRET with an electron-multiplying charge-coupled device (EMCCD) camera as a detector is limited by several factors, such as photobleaching and photoblinking of the fluorophore and low photon count rate. Because of this low temporal resolution, EMCCD-based immobilized smFRET is not optimal for studying biomolecular dynamics on a timescale shorter than ∼10 ms (ref. 14). Because of this technical limitation, processes with millisecond-scale dynamics, such as conformational changes in machinery proteins, protein diffusion on DNA, protein folding and the gating of ion channels, have not yet been actively investigated using smFRET^14^,^15^,^16^,^17^. As a result, technical developments that will permit the monitoring of fast dynamics are in high demand in the single-molecule field.

The broadness of the FRET distribution relative to that of a shot-noise-limited FRET distribution has been used to analyse the dynamics at the millisecond scale, for example, in probability distribution analysis^18^,^19^ and proximity ratio histogram analysis^20^. The burst variance analysis method analyses the fluctuations over time for individual single molecules, and this analysis discriminates between static and dynamic fluctuations^21^. However, these methods cannot directly observe the real-time FRET change in a time trace to identify discrete FRET states and the transition rates in biomolecules, as can be performed using immobilized smFRET. Recently, a line confocal excitation has been applied to a flowing sample; however, the observation duration was not sufficiently long to resolve the transition dynamics^22^. An anti-Brownian electrokinetic trap can be used to observe a molecule in solution for 1 s; however, the FRET dynamics at the millisecond scale has not yet been demonstrated with a high photon-counting rate^23^,^24^.

Here, we report a simple but direct method of monitoring the submillisecond-scale dynamics of a single molecule by measuring the FRET of a single diffusing molecule in a buffer solution for tens of milliseconds with submillisecond temporal resolution. To achieve a long observation time with the enhanced temporal resolution, we tether the target biomolecules to freely diffusing liposomes and then apply a photoprotection buffer (Fig. 1a). A high signal-to-noise ratio and a high photon-counting rate are maintained in this method by means of applying confocal excitation and a highly sensitive avalanche photodiode (APD) with a pinhole for the measurements in the buffer solution. Using this approach, we obtain FRET time traces for fluctuations between two conformational states of the Holliday junction (HJ) and measure HJ conformational dynamics with transition rates as high as 1,500 s^−1^ (∼0.7-ms average dwell time). Then, we further demonstrate the application of this technique to DNA–protein interactions by directly monitoring the diffusional migration dynamics of the single-stranded (ss) DNA-binding proteins (SSBs) from *Deinococcus radiodurans* (*dr*) and *E. coli* on ssDNA. The transition rate of *dr*SSB is ∼200 s^−1^ (5-ms dwell time) at room temperature, which is more than an order of magnitude faster than that of *E. coli* SSB. Thus, we expect that the approach presented in this work will become a powerful tool for investigating the submillisecond dynamics of biomolecules.

## Results

### Extending the observation time by liposome tethering

**Figure 1:**
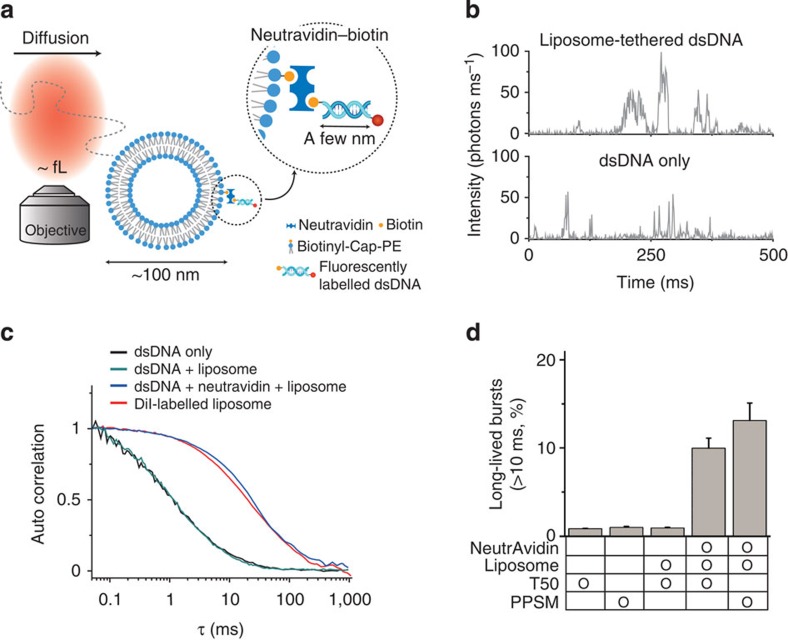
Extending the observation duration using liposome tethering and photoprotection. (**a**) Schematic description of the liposome-tethering approach. Tethering of the FRET molecule to a liposome is achieved through the biotin–NeutrAvidin (NA) interaction. (**b**) Typical time traces of fluorescently labelled dsDNA with liposome tethering (upper panel) and without liposome tethering (lower panel). (**c**) Autocorrelation curves measured using FCS. The fluorescently labelled dsDNA only (black line) and the fluorescently labelled dsDNA in the presence of liposomes without NA (green line) exhibited rapid diffusion with an average transit time of ∼1 ms. In the presence of both NA and liposomes (blue line), the transit time of the fluorescently labelled dsDNA increased to ∼20 ms, which is comparable to that of the liposome itself (red line). (**d**) Bar graph of the fraction of long-lived single bursts (>10 ms) with or without liposome tethering. T50 represents a buffer containing 10 mM Tris-HCl (pH 8.0) and 50 mM NaCl, and photo-protection single-molecule buffer (PPSM) is a buffer composed of T50+10 mM cysteamine and 1 mM Trolox. The error bars denote the s.d.'s obtained from three independent measurements.

Because EMCCD-based immobilized smFRET has limited temporal resolution^5^, APD-based smFRET is often more practical for measuring the fast dynamics of a biomolecule at submillisecond resolution. Indeed, APDs have been used for smFRET on surfaces to detect the fast dynamics of biomolecules^25^,^26^,^27^. However, the detection throughput is limited when APDs are used to investigate samples that are immobilized on a glass surface because the specific location of each molecule on the stage must be determined for each measurement. In contrast to the confocal detection of a single molecule immobilized on the glass surface, diffusion-based smFRET using an APD detects single molecules with high throughput at a given time without the need for surface immobilization^7^,^8^,^9^,^10^,^11^,^28^. However, diffusion-based smFRET suffers from a short observation time^7^,^8^. The transit time of biomolecules, such as proteins and DNA, through the confocal excitation area is typically ∼1 ms (Fig. 1a), and thus, a time trace obtained using diffusion-based smFRET rarely lasts longer than a few milliseconds^16^. Because of these short time traces, diffusion-based smFRET has mostly been used for subpopulation analysis rather than for the observation of the real-time dynamics of biomolecules^7^,^8^,^9^,^10^,^29^.

Although DNA and proteins have sizes on the order of a few nanometres, liposomes, that is, vesicles composed of various phospholipids, can range in size from tens to hundreds of nanometres^30^. Thus, the diffusion of a liposome is much slower than that of typical biomolecules, and its time trace lasts for tens of milliseconds^30^. The size of a liposome can be varied easily by controlling the size of the membrane pores during the liposome preparation step. More importantly, the optical transparency of liposomes in the visible range induces a negligible level of background signal (Supplementary Fig. 1). By incorporating F_0_F_1_-ATP synthase into liposomes, Börsch and colleagues have observed the conformational change in F_0_F_1_-ATP synthase in solution by increasing the dwell time of the diffusing molecule^31^,^32^. However, this approach can only be applied for membrane proteins that can be embedded into a lipid bilayer; it cannot be used to study the dynamics of other soluble biomolecules, such as nucleic acids and cytosolic proteins.

Here, we employed a tethering approach, in which target biomolecules were tethered to freely diffusing liposomes of 100 nm in diameter through the biotin–NeutrAvidin interaction (Fig. 1a). Because the diffusion coefficient is inversely proportional to the size (hydrodynamic radius) of a diffusing particle, according to the Stokes–Einstein equation, the tethering of a biomolecule to a liposome slows its diffusion, thereby extending the duration of a single burst (Fig. 1b). We obtained the effective confocal volume of our set-up using fluorescence correlation spectroscopy (FCS) and well-known diffusion coefficient of rhodamine 6G, which was ∼1 fl. Then, we converted the diffusion time into the diffusion coefficient of the liposome*, D*_liposome_∼3.0 μm^2^ s^−1^, which is ∼15-fold smaller than that of 40 base-pair DNA oligomers (46.4 μm^2^ s^−1^). To ensure that a liposome attached no more than one fluorescently labelled dsDNA, we used a 100-fold molar excess of the vesicle and NeutrAvidin compared with the fluorescently labelled dsDNA (Supplementary Discussion). FCS measurements with and without liposome tethering were performed to quantitatively verify that tethering extended the measurement time (Fig. 1c). As expected, without liposome tethering, the diffusion time of the dsDNA (40 bases) was 1.3 ms. Liposome tethering increased the diffusion time of the dsDNA to ∼20 ms, which is similar to the diffusion time of a liposome labelled with the fluorescent lipid 1,1'-dioctadecyl-3,3,3'3'-tetramethylindocarbocyanine perchlorate (Fig. 1c). When dsDNA was mixed with liposomes without NeutrAvidin, no change in the diffusion time of the dsDNA was observed (Fig. 1c). Thus, the increase in the diffusion time of the dsDNA was caused by the liposome tethering.

Next, we analysed the fraction of long-lived single bursts (Fig. 1d). We counted the number of bursts that had a duration longer than 10 ms (1-ms binning with a threshold of 30 photons per ms; Fig. 1d). In the case of the dsDNA without liposome tethering, less than 1% of bursts had a burst duration longer than 10 ms, and burst durations longer than 20 ms were not observed at all. With liposome tethering, the fraction of bursts that lasted longer than 10 ms increased to ∼10 % of the total (Fig. 1d). Thus, the liposome-tethering method increased the number of long-lived bursts at a given time, which enabled proper statistical analysis with a sufficient number of time traces in addition to simply providing observations of longer burst duration for single diffusing molecules.

### Enhancing the temporal resolution by high photon count rate

Extension of the observation duration in diffusion-based smFRET does not ensure a high temporal resolution in single-molecule FRET: a high photon-counting rate is necessary to achieve a high temporal resolution. The photon-counting rate in diffusion-based smFRET is typically ∼30 photons per ms under conventional conditions^7^,^8^,^20^. A higher photon-counting rate can be achieved by increasing the power of the excitation laser; however, in this case, the photon-counting rate is limited by the photobleaching and blinking of the probe dyes^33^,^34^; these photophysical phenomena reduce the observation duration for a single diffusing molecule (Supplementary Fig. 2a). Munoz and colleagues^35^, however, have reported that the use of a mixture of cysteamine and Trolox reduces the photobleaching and blinking rates and thus enhances the emission rate, even under high excitation power. The authors achieved a photon-counting rate of up to ∼500 counts per ms using diffusing molecules in a buffer solution^35^.

Thus, we applied a cysteamine-Trolox mixture in our liposome-tethering method to enhance the photon-counting rate. In the presence of 10 mM cysteamine and 1 mM Trolox, a photo-protection single-molecule buffer, the amount of long-lived single-molecule bursts were maintained even at a laser power of 130 kW cm^−2^ (Supplementary Fig. 2a). Importantly, the number of bursts with high photon-counting rates (>200 photons per ms) was significantly increased by a factor of eight (Supplementary Fig. 2b). As a consequence, combining the liposome-tethering approach with the photo-protection buffer allowed us to increase the fraction of single-molecule fluorescence time traces that were longer than 10 ms up to 13% (the longest single time traces were ∼100 ms; Figure 1d) and to increase the photon-counting rate to ∼400 photons per ms in solution (Supplementary Fig. 2); these conditions are sufficient for the study of millisecond-scale dynamics in biomolecules with a temporal resolution of ∼0.2 ms.

### Real-time dynamics of HJ at submillisecond resolution

**Figure 2:**
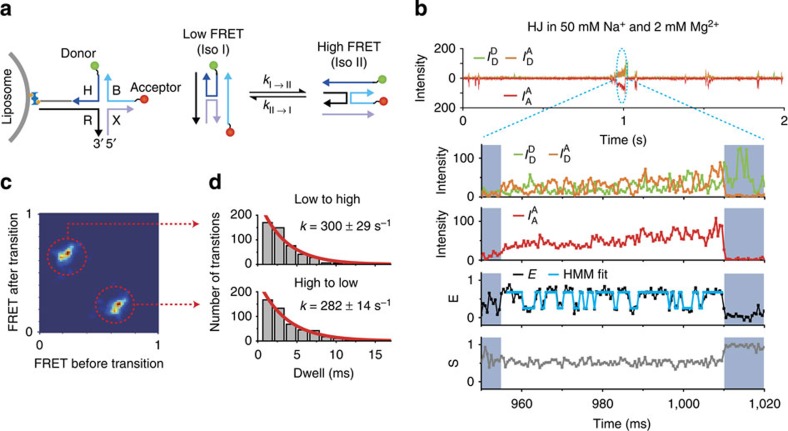
Observing the real-time dynamics of FRET with submillisecond resolution. (**a**) Schematic illustrations of the liposome tethering of a HJ and the conformational transition of the HJ. (**b**) Typical time trace of the HJ dynamics at 50 mM NaCl and 2 mM MgCl_2_. In ALEX mode, three intensities, 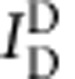 (green line), 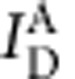 (orange line) and 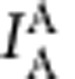 (red line), were obtained as raw data, where 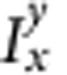 denotes the emission from the *y* molecule caused by the *x* molecule excitation. The FRET efficiency *E* (black line) and stoichiometry *S* (grey line) were calculated for each time bin (0.5 ms). The blue line represents the fit to the FRET efficiencies obtained using hidden Markov modelling. (**c**) Transition-density plot of the HJs at 2 mM MgCl_2_ measured using the liposome-tethering approach (180 molecules). (**d**) Dwell-time distributions of conformer transitions, *Iso I* to *Iso II* (low *E* to high *E*, upper panel) and *Iso II* to *Iso I* (high *E* to low *E*, lower panel); the single-exponential fits reveal transition rates of 300±29 and 282±14 s^−1^, respectively.

To demonstrate a real-time measurement of single-molecule dynamics within a single burst using the liposome-tethering method, we measured the dynamics of the HJ, an intermediate in the genetic recombination process^36^, whose conformational dynamics has been extensively studied using immobilized smFRET^37^,^38^,^39^. We prepared HJs labelled with Atto-550 as a FRET donor and Atto-647N as a FRET acceptor, and we then attached each HJ to a liposome using the NeutrAvidin–biotin interaction (Fig. 2a). The conformer dynamics between two stacked conformers of HJ was directly observed within a single burst using this tethering approach (Fig. 2b). We applied alternating-laser excitation (ALEX) to detect single molecules in solution; ALEX uses two lasers, one each for the donor and acceptor excitations, that are switched on a timescale shorter than the transit time of a molecule^8^,^40^. Using ALEX is particularly significant in the study of fast dynamics because the ability to directly detect the acceptor intensity simultaneously with the donor intensity and the FRET-induced acceptor intensity makes it possible to monitor the artefacts caused by photophysical effects, such as blinking, photobleaching or spectral shifts^34^. ALEX determines the FRET efficiency (***E***, a measure of the proximity between two dyes) and the stoichiometry parameter (***S***, a measure of the labelling status of a molecule with donor and acceptor dyes) from the ratios of three types of emissions: 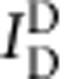 is the fluorescence emission of the donor dye excited by the donor-excitation laser (532 nm), 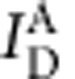 is the fluorescence emission of the acceptor dye excited by the donor-excitation laser (532 nm) and is a FRET signal, and 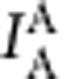 is the fluorescence emission of the acceptor dye excited by the acceptor-excitation laser (633 nm). Figure 2b presents the fluorescence time traces of a HJ tethered to a freely diffusing liposome (0.5-ms binning), which lasted ∼53 ms in solution. The FRET time trace clearly illustrates the transitions between two conformer states of the HJ (***E***=0.25 for *Iso I* and ***E***=0.75 for *Iso II*)^37^,^38^,^39^ at the submillisecond timescale. The stoichiometry (***S***) time trace (Fig. 2b, bottom panel), a unique parameter available in the ALEX mode, is used to monitor photophysical artefacts, such as blinking, spectral shifts of the acceptor or photobleaching, which result in sudden alterations in ***S***. For example, the sudden increment in ***S*** at ∼1,010 ms indicates photobleaching of the acceptor dye (Fig. 2b). When ***S*** varied significantly during a burst time trace except for the permanent photobleaching, we removed that time trace from the analysis. However, these bursts occurred rarely in the HJs (<1% of total time traces), which indicates that the potential spectral shift or blinking of Atto-647N is not a significant factor in our measurement conditions^34^. We confirmed that the high photon-count rate does not distort the FRET distribution by comparing FRET distributions obtained at low and high photon count rates using a HJ (Supplementary Fig. 3). We also confirmed that the possible orientational fluctuations of probe dyes did not cause the FRET fluctuation of HJ (Supplementary Fig. 4 and Supplementary Discussion).

Then, we used hidden Markov modelling (HMM) to determine the number of FRET states and to calculate the transition rates between the states using vbFRET (Fig. 2b, blue line)^41^,^42^. vbFRET using variational Bayesian expectation maximization^41^ is more accurate than the maximum likelihood methods when analysing fast transitions: vbFRET determined the transition rates of synthetic FRET traces with >90% accuracy (Supplementary Fig. 5, Supplementary Fig. 6 and Supplementary Table 1). Thus, we analysed individual time traces that were longer than 15 ms (180 individual molecules) using the vbFRET analysis. The result presented in the transition-density plot (Fig. 2c) clearly indicates two states, which gave highest maximized evidence^41^. The transition rates between the two states at 50 mM NaCl and 2 mM MgCl_2_ were calculated from the dwell-time distribution of each transition (Fig. 2d): 300±29 s^−1^ for low ***E*** to high ***E*** and 282±14 s^−1^ for high ***E*** to low ***E***. These results are consistent with previous results obtained via cross-correlation analysis with 2-ms time resolution using immobilized smFRET^42^. The HJ activation energy measured using our approach was also consistent with these previous measurements (Supplementary Fig. 7). These results indicate that the liposome tethering had a negligible effect on the HJ dynamics. When using immobilized smFRET, it is not possible to directly observe the rapid transitions between states that are apparent in the FRET time trace shown in Fig. 2b.

### Minimum number of transitions for quantitative analysis

For the analysis of transition rates using HMM, the use of time traces whose durations are sufficiently long relative to the transition rate is vital for quantitative measurement^42^. When the time trace of a molecule is not sufficiently long to display all transitions, HMM analysis cannot properly determine the discrete states in a molecule because some states are missing from the trace. In such a case, several individual time traces can be stitched into a single time trace to increase the number of data points for the HMM analysis and thus permit the analysis to identify all discrete states^43^,^44^. However, the effect of stitching together multiple time traces for the HMM analysis on the transition rates thus obtained has not been previously examined. Therefore, we carefully tested the effect of stitching together multiple time traces using HJ time traces. We varied the number of stitched time traces from 1 to 10, using traces longer than 15 ms (3-ms average dwell time), to check whether the process of stitching analysis causes any artefact or not. No differences were observed in the number of discrete states and the transition rates between the states that were obtained from the analyses of the stitched time traces (Supplementary Fig. 8).

**Figure 3:**
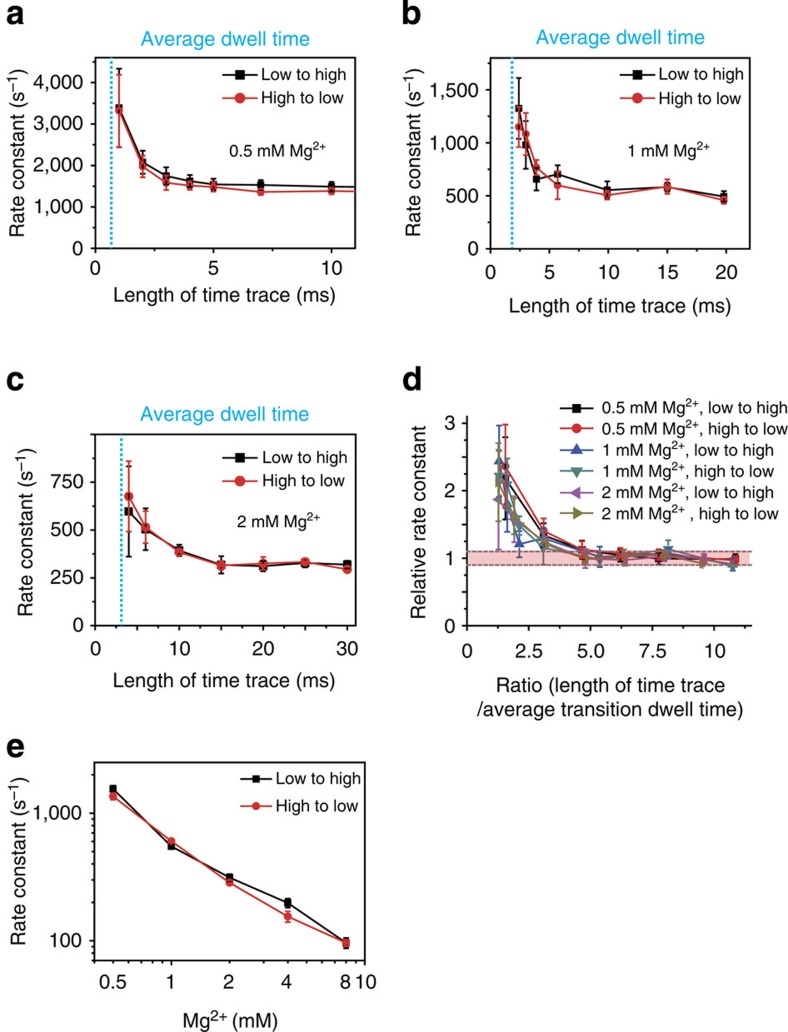
Minimum number of transitions within a burst necessary for quantitative analysis. (**a–c**) The transition rates calculated via the HMM analysis for HJ time traces of various lengths at (**a**) 0.5 mM MgCl_2_, (**b**) 1 mM MgCl_2_ and (**c**) 2 mM MgCl_2_. The blue dashed lines represent the average dwell time of the HJs in each case. (**d**) Comparison of the dependence of the rate constant on the ratio of the original time-trace length to the dwell time for various transitions. The ratio (*x* axis) is calculated by dividing the length of the original time traces by the average dwell time for the transition. The relative rate constant (*y* axis) is calculated by dividing the rate constant obtained from the stitched time trace by the rate constant obtained from a time trace that is sufficiently long for the HMM analysis without being stitched together from shorter traces. (**e**) Dependence of the HJ transition-rate constant on the Mg^2+^ concentration. The error bars denote the fitting uncertainty of the exponential fit to dwell-time distributions. The error bars in **e** are smaller than the symbol size.

We then analysed the minimum length of the original time traces required to quantitatively measure the transition rate using stitched time traces (Fig. 3). We prepared time traces for this investigation by intentionally shortening them; the traces were cut at specific points to obtain time traces of given lengths (*x* axis in Fig. 3a–c), and we then stitched the time traces together to produce traces of ∼100 ms in length and finally applied the HMM analysis to the stitched traces. When the length of the original time traces was comparable to the average dwell time, the transition rate of the 100-ms stitched time trace was typically overestimated by the HMM analysis (Fig. 3a–c). However, as the length of the original time traces increased, the accuracy of the analysis improved. In our measurement, time traces longer than the average transition dwell time by approximately a factor of four permitted the analysis to yield transition rates with less than 10 % error once they were stitched together (Fig. 3d).

### Dynamic temporal range of the liposome-tethering approach

Using the criteria detailed in Fig. 3d, the transition rates of the HJs at various Mg^2+^ concentrations were measured to address the limits of the temporal resolution and the dynamic range of our method (Fig. 3e). A transition rate of ∼1,500 s^−1^ (∼0.7-ms average dwell time) was the limit of our approach because of the photon-counting rate. For slow transitions, our method can measure a rate of as low as 100 s^−1^ (10-ms average dwell time) because of the limitation on the observation duration (time-trace length). EMCCD-based immobilized smFRET typically permits the measurement of transitions with rates slower than ∼100 s^−1^ (10-ms dwell time); thus, our approach successfully addresses the dynamic range that is difficult to be explored using EMCCD-based immobilized smFRET, the range of transition rates between 100 and 1,500 s^−1^ (dwell times of 0.7–10 ms).

### Application to *dr*SSB migration on ssDNA

Next, we applied our approach to the study of the millisecond-scale dynamics of protein–DNA interactions using SSB migration on ssDNA as a model system. By protecting ssDNA from degradation and recruiting other DNA-maintenance proteins to act on ssDNA, SSB plays essential roles in replication, recombination and DNA repair in cells^45^. In previous work, the one-dimensional migration of *E. coli* SSB on ssDNA with a discrete step size, which is termed ‘diffusional migration', has been investigated using immobilized smFRET^46^,^47^. However, because immobilized smFRET has a limitation in its temporal resolution, the diffusional migration dynamics of *E. coli* SSB faster than ∼20-ms dwell time (50 s^−1^ transition rate) could not be observed, such as the transition at 37 °C.

**Figure 4:**
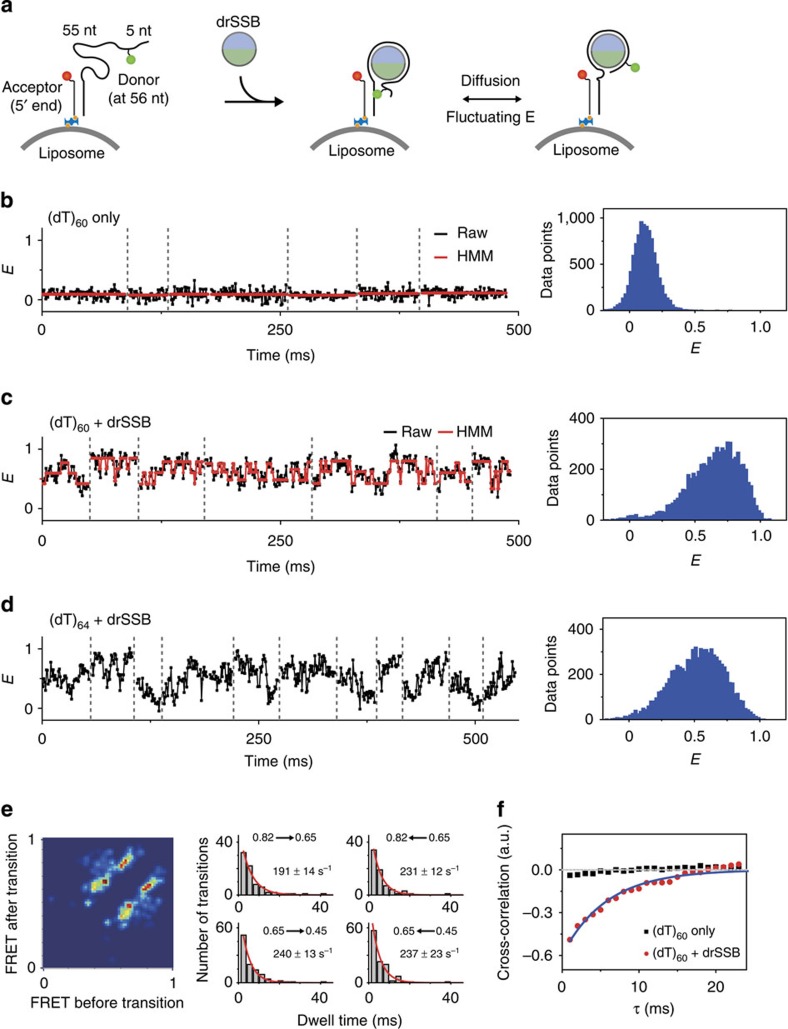
*D. radiodurans* SSB diffusion on ssDNA. (**a**) Schematic representation of the experiments for *dr*SSB diffusion on ssDNA. Dye-labelled DNA containing a single-stranded overhang was tethered to liposomes using NeutrAvidin. The *dr*SSB wrapped the ssDNA and then diffused on the ssDNA, which resulted in fluctuations in the FRET efficiency. (**b**,**c**) FRET time traces and FRET histograms of (dT)_60_ at room temperature: (**b**) without *dr*SSB (six molecules are presented among total 251 molecules) and (**c**) with *dr*SSB (seven molecules are presented among total 168 molecules). The red lines represent the fits obtained using HMM. (**d**) FRET time trace and FRET histogram of (dT)_64_ with *dr*SSB (eleven molecules are presented among total 124 molecules). (**e**) Transition-density plot and stepping rates of (dT)_60_ with *dr*SSB at room temperature. (**f**) Cross-correlation analysis for *dr*SSB diffusion. The average cross-correlation curves were obtained from the time traces of (dT)_60_ with *dr*SSB (red circle) and without *dr*SSB (black square). The blue line on the cross-correlation plot represents single-exponential fit with apparent rate of *dr*SSB diffusion, 172±45 s^−1^.

Here, we investigated the migration dynamics of *dr*SSB and *E. coli* SSB on ssDNA. *dr*SSB is a homodimer that belongs to the *Deinococcus*/*Thermus* group of SSBs^40^,^48^. It has been reported that a *dr*SSB dimer occludes 50–55 nucleotides (nt) of poly(dT) at high salt concentrations (≥200 mM NaCl), which is 8–13 nt shorter than for homotetrameric *E. coli* SSB^49^,^50^. The binding energy of *dr*SSB to ssDNA, 94 kcal mol^−1^, is smaller than that of *E. coli* SSB, 130 kcal mol^−1^ (ref. 49). Thus, we expected the migration speed of *dr*SSB to be faster than that of *E. coli* SSB. We used our tethering approach at room temperature to investigate this fast dynamics (Fig. 4a). When ssDNA alone without *dr*SSB was tethered to a liposome, an average FRET of 0.12 with no fluctuation was observed (Fig. 4b). For the (dT)_60_ sample, an increased FRET was expected from the *dr*SSB–ssDNA structure, as *dr*SSB wraps ssDNA^51^. Indeed, (dT)_60_-*dr*SSB presented a FRET of ∼0.65 at a high salt concentration (200 mM NaCl). Notably, considerable FRET fluctuation can be observed in this time trace with millisecond binning (Fig. 4c). The transition-density plot of (dT)_60_-*dr*SSB, obtained by vbFRET, exhibits two major steps (Fig. 4e). To confirm that the fluctuation in FRET was induced by *dr*SSB diffusional migration, we elongated the ssDNA by 4 nt to (dT)_64_, which was expected to increase the FRET fluctuation range by increasing the migration range of *dr*SSB on ssDNA (Fig. 4d). As anticipated, the FRET of (dT)_64_-*dr*SSB fluctuated more broadly, with an average FRET of 0.51. These results indicate that *dr*SSB has diffusional migration on ssDNA, similarly to *E. coli* SSB. The stepping rate of *dr*SSB on (dT)_60_ was 190–240 s^−1^, as measured from the transition-density plot, which is consistent with the result of the cross-correlation analysis (Fig. 4f). The transition states of (dT)_64_ are not clearly separated from each other in the transition-density plot; however, the transition rate measured from the cross-correlation was similar to that measured for (dT)_60_ (Supplementary Fig. 9). We note that the diffusional stepping rate of *dr*SSB is an order of magnitude higher than that of *E. coli* SSB reported in ref. 46 at room temperature. The activation energy of *dr*SSB diffusional migration was measured from the Arrhenius plot and was found to be 28±7 kJ mol^−1^ (Supplementary Fig. 10). This value is approximately one-third of the activation energy of *E. coli* SSB (81 kJ mol^−1^)^46^, which may explain the rapid migration of *dr*SSB compared with *E. coli* SSB.

**Figure 5:**
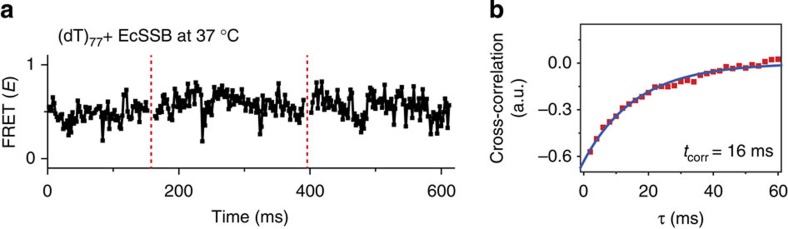
Direct observation of the diffusional migration of *E. coli* SSB at 37 °C. For the measurement of *E. coli* SSB migration on ssDNA, we mixed 1 nM (dT)_77_ with 100-fold excess of NeutrAvidin and biotinyl liposome in a T200 buffer (10 mM Tris-HCl pH 8.0 and 200 mM NaCl). Liposome-tethered (40 pM) (dT)_77_ in the presence of 2 nM *E. coli* SSB in a T200 buffer with 1 mM cysteamine and 10% glycerol was used for ALEX measurement at 37 °C. (**a**) Typical time traces of *E. coli* SSB diffusional migration on ssDNA. (**b**) Correlation analysis of *E. coli* SSB diffusional migration.

For the comparison of the diffusional rates between *dr*SSB and *E. coli* SSB, the diffusional rate of *E. coli* SSB on (dT)_77_ was measured at 37 °C using our tethering method (Fig. 5). The transition time of *E. coli* SSB migration was ∼16 ms (∼63 s^−1^ transition rate), which is in excellent agreement with the estimation of previous work (∼60 s^−1^)^46^. The transition time of *E. coli* SSB migration at 37 °C is more than four times slower than that of *dr*SSB (∼3.6 ms) at 37 °C, calculated from the Arrhenius plot in Supplementary Fig. 10b, and even much slower than that of *dr*SSB at 31 °C, ∼4.3 ms. Thus, our results obtained using liposome tethering clearly demonstrate that the diffusional migration speed of *dr*SSB is much faster than that of *E. coli* SSB.

## Discussion

In this study, we demonstrated the direct measurement of single-molecule FRET dynamics with submillisecond temporal resolution in aqueous solution using liposome tethering. The single-molecule dwell time in the excitation volume was increased by more than 20-fold without sacrificing the high signal-to-noise ratio and high photon-detection efficiency of confocal excitation^52^. Because our approach is a diffusion-based single-molecule measurement, complicated and time-consuming surface-coating steps for the preparation of quartz slides, which typically require overnight incubation, are not necessary. Liposome tethering requires a simple 10-min incubation of the mixture. This procedure is also simpler than the time-consuming procedures for proteoliposome reconstitution required by a previous method, which include detergent breakdown, dialysis and the further purification of proteoliposomes from free proteins^30^,^31^,^32^,^53^. Encapsulation of a FRET pair in a vesicle has been used for tethering-free assay^54^. However, this method requires high concentration of FRET samples for encapsulation in vesicles, and it is difficult to change buffer condition once FRET samples are encapsulated in vesicles.

One of the advantages of our method is that it achieves measurements of real-time submillisecond dynamics without the need for any additional sophisticated apparatus. Other methods that can bypass the need for the glass-surface immobilization of samples require the addition of sophisticated devices, such as microfluidic devices^55^, electrokinetic traps^24^ or capillary cells^52^, to the basic microscopy set-up for single-molecule detection. Unlike other techniques, our approach requires only the preparation of simple vesicles and uses the basic microscopy set-up for single-molecule detection.

Currently, we could obtain more than 200 molecules of time traces that are longer than 10 ms with submillisecond temporal resolution in 10 min measurement. Combining our method with the multichannel confocal detection scheme that has been developed by Weiss and colleagues^56^ will increase the throughput and thus permit the measurement of a larger number of long time traces at a given time. Most single-molecule studies using glass-surface-immobilized smFRET have used avidin–biotin interactions to attach molecules to a surface^57^. As a consequence, the use of NeutrAvidin–biotin interactions for tethering in this work allows for the immediate application of our approach to other biological systems that undergo millisecond dynamics and have been studied using immobilized smFRET. Although the dynamic range of our approach is currently 100 s^−1^–1,500 s^−1^, the application of more photostable organic dyes, such as self-healing fluorophores^33^, will further increase both its temporal resolution and the observation duration for FRET of our method.

Recently, Ha and colleagues^46^,^58^ have studied the dynamics of *Thermus thermophilus* (*tt*) SSB on ssDNA. *tt*SSB is a homodimeric SSB similar to *dr*SSB. They have reported that the diffusional dynamics of *tt*SSB on ssDNA, which was composed of polyT, was too fast to be monitored using the 30-ms temporal resolution of immobilized FRET at room temperature under high-salt conditions; they could observe the diffusional dynamics of *tt*SSB at intermediate-salt concentration. However, we could measure the transition time of *dr*SSB, another type of homodimeric SSB, using polyT ssDNA at room temperature under high-salt conditions, which was ∼5 ms. These results show that our method would be very useful for investigating fast dynamics of homodimeric SSBs. In addition, our results together with previous work by Ha and colleagues^46^,^58^ indicate that the diffusional migration or redistribution of SSB on ssDNA is a general phenomenon. The diffusional migration of *dr*SSB is much faster than that of tetrameric *E. coli* SSB. The fast migration and low binding energy of *dr*SSB should accelerate *dr*SSB's redistribution on ssDNA and removal from ssDNA for downstream DNA repair processes, compared with those of *E. coli* SSB. This may provide one of the clues for the explanation why *D. radiodurans* has a high tolerance to the DNA-damaging environment^59^.

## Methods

### dsDNA and HJ preparation

To test the liposome-tethering approach and its effect on the observation duration, we prepared dsDNA that contained both biotin and fluorescent dye. The sequences were 5'-(Atto-647N-T)AAATCTAAAGTAACATAAGGTAACATAACGGTAAGTCCA-Biotin-3'. The dsDNA was hybridized via the heating and slow cooling in T500 buffer (10 mM Tris-HCl and 500 mM NaCl). The amount of unlabelled complementary oligonucleotide that was used was 1.5-fold greater than the amount of labelled oligonucleotide to ensure complete annealing of the fluorescently labelled oligonucleotide on the dsDNA. The following sequence was used for the oligonucleotides for the HJs: H strand, 5'-Atto-550-CCGTAGCAGCGCGAGCGGTGGG; B strand, 5'-Atto-647N-CCCTAGCAAGCCGCTGCTACGG; X strand, 5'-CCCAGTTGAGCGCTTGCTAGGG-3'; R strand, 5'-TGGCGACGGCAGCGAGGAATACCCACCGCTCGGCTCAACTGGG; and biotin strand, 5'-TATTCCTCGCTGCCGTCGCCA-biotin-3'. The oligonucleotide strands were mixed in a H:B:X:R:biotin-strand ratio of 1:1:2:2:4 for hybridization. The mixture was heated at 90 °C for 2 min and then slowly cooled to room temperature in a water bath for ∼5 h.

### SSB preparation

The *D. radiodurans* ss binding protein (*dr*SSB) gene cloned into a pET21a vector was expressed in BL21(DE3) in N-terminal His6-tag form and purified at high affinity using an Ni-NTA Superflow (Qiagen). The *dr*SSB was eluted with a buffer that contained 500 mM imidazole. We performed size-exclusion column chromatography using a PD minitrap G-25 (GE Healthcare) to remove the imidazole from the eluate. The *dr*SSB was stored in a storage buffer (Tris-HCl pH 8.3, 500 mM NaCl, 20 % glycerol (v/v) and 1 mM dithiothreitol) at −80 °C. *E. coli* SSB was purchased from PROMEGA (Catalogue number: M301A).

### Liposome preparation

Synthetic lipids, 1-palmitoyl-2-oleoyl-sn-glycero-3-phosphocholine (POPC), 1,2-dioleoyl-sn-glycero-3-[phospho-L-serine] (DOPS), 1-palmitoyl-2-oleoyl-sn-glycero-3-phosphoethanolamine (POPE), cholesterol and 1,2-dipalmitoyl-sn-glycero-3-phosphoethanolamine-N-(cap biotinyl) (Biotinyl Cap PE) were mixed together at the desired molar ratio (POPC:DOPS:POPE:Chol:Biotinyl Cap PE=54.9:5:20:20:0.1). The mixture of lipids was dried and kept in vacuum for at least 5 h to ensure the thorough removal of residual chloroform. The dried lipid film was hydrolysed with T50 buffer (10 mM Tris-HCl, pH 8.0, 50 mM NaCl) through rigorous vortexing. Afterwards, a cycle of freezing and thawing in liquid nitrogen followed by a water bath (35 °C) was repeated more than 10 times to form large unilamellar vesicles. To prepare monodisperse unilamellar liposomes, we performed extrusion using a mini extruder (Avanti Polar Lipid) with a 100-nm polycarbonate filter (Whatman). The final concentration of the liposome was 10 mM in lipid concentration, which corresponds to ∼150 nM in liposome units. The size of the liposomes was confirmed via a dynamic light-scattering measurement (Supplementary Fig. 11).

### Alternating-laser-excitation for HJ and *dr*SSB

The microscope set-up for ALEX has been extensively described elsewhere^30^, and its modification is described in detail in the Supplementary Methods. For the HJ dynamics measurement, we incubated 1 nM HJ and 100 nM NeutrAvidin for 5 min; 100 nM biotinyl liposome was then added to the mixture, which was then incubated in a T50 buffer (10 mM Tris-HCl pH 8.0 and 50 mM NaCl). To achieve single-molecule detection in the ALEX measurement, we diluted the mixture to a 40-pM concentration of HJ with photoprotection single-molecule buffer (T50 buffer + 10 mM cysteamine + 1 mM Trolox). Fluorescence bursts in the time trace caused by events involving the inward and outward diffusion of liposome-tethered HJs were obtained in the raw data from the ALEX measurement. By alternating two lasers, three different types of photons were obtained for each burst: 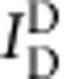 is the fluorescence emission of the donor dye (Atto-550) excited by the donor-excitation laser (532 nm), 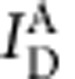 is the fluorescence emission of the acceptor dye (Atto-647N) excited by the donor-excitation laser (532 nm) and is a FRET signal and 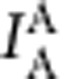 is the fluorescence emission of the acceptor dye (Atto-647N) excited by the acceptor-excitation laser (633 nm). The start and end times of each burst were determined using the dual-channel burst-search method, which uses two thresholds simultaneously; one is 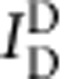
+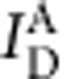
>30 photons, and the other is 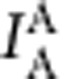
>30 photons per binning time. We used 20 photons as the threshold value for 0.2-ms binning. The dual-channel burst-search finds only dually labelled HJ and eliminates potential artefacts caused by photobleaching and photoblinking^20^. For *dr*SSB diffusion, we mixed 1 nM (dT)_60_ with 100-fold excess of NeutrAvidin and biotinyl liposome in a T200 buffer (10 mM Tris-HCl pH 8.0 and 200 mM NaCl). Liposome tethered (40 pM) (dT)_60_ in the presence of 100 nM *dr*SSB in a T200 buffer with 1 mM cysteamine was used for ALEX measurement.

### HMM and stitching time traces

Before the HMM analysis, 5–10 traces of single diffusing molecules were stitched together to ensure that there was a sufficient number of data points for the HMM fit (100–200 data points)^42^. The stitched traces were analysed using HMM, and they were then separated into the original single traces to eliminate nonphysical transitions between different time traces introduced by the stitching procedure. Transition-density plots and dwell times were also obtained from the individual separated traces. The HMM analysis is described in detail in the Supplementary Method.

### Cross-correlation analysis

The rates of FRET fluctuation caused by *dr*SSB diffusion at various temperatures were obtained from the cross-correlation between the donor-intensity 
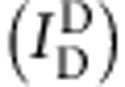
 and acceptor-intensity 
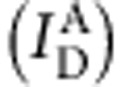
 time traces. To eliminate the effect of the total intensity fluctuation in the diffusion-based measurement, we normalized the donor and acceptor intensities by the total intensity of the donor-excitation laser 
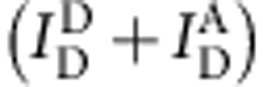
. For each measurement, the cross-correlation curves were averaged over 50 traces; only traces longer than 25 ms were used. The correlation time, *τ*, was deduced by fitting the cross-correlation curve to a single exponential function, *e*^−*t*/*τ*^. More than four measurements were performed at each temperature.

## Author contributions

J.-Y.K. and N.K.L. designed the experiments; J.-Y.K. and N.K.L. wrote the manuscript; J.-Y.K. and C.K. performed sample preparation, measurements and analysis.

## Additional information

**How to cite this article:** Kim, J.-Y. *et al*. Real-time submillisecond single-molecule FRET dynamics of freely diffusing molecules with liposome tethering. *Nat. Commun*. 6:6992 doi: 10.1038/ncomms7992 (2015).

## Supplementary Material

Supplementary Information Supplementary Figures 1-11, Supplementary Table 1, Supplementary Discussion, Supplementary Methods and Supplementary References
