# Supplementary material for: Real-time submillisecond single-molecule FRET dynamics of freely diffusing molecules with liposome tethering
Source: Nat Commun. 2015 Apr 24;6:6992. doi: 10.1038/ncomms7992 (PMC4421855; doi:10.1038/ncomms7992)
Supplement: Supplementary Information — Supplementary Figures 1-11, Supplementary Table 1, Supplementary Discussion, Supplementary Methods and Supplementary References [file ncomms7992-s1.pdf]

Supplementary Figures

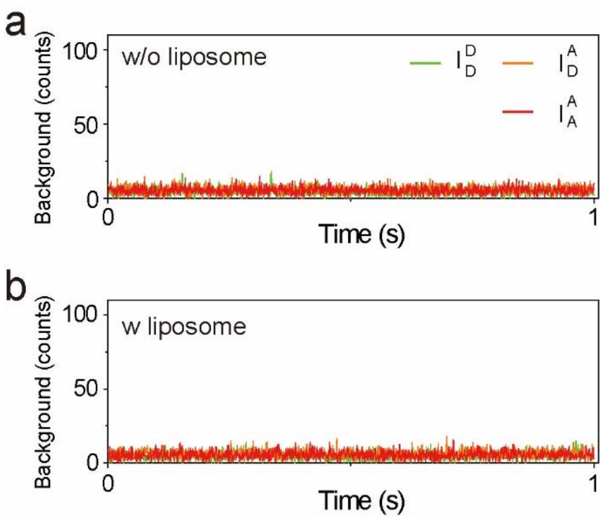

Supplementary Fig. 1. Comparison of background levels of single-molecule measurement. (a-b) Typical levels of background signals of buffer solution at the power of 1 mW for 532 nm and 0.3 mW for 633 nm lasers in alternating mode. The excitation lasers were focused at a point 20 μm from the surface of a coverslip in solution: (a) without liposome and (b) with 500 μM liposome in lipid concentration. No difference in the background signals was observed with and without liposome in solution.

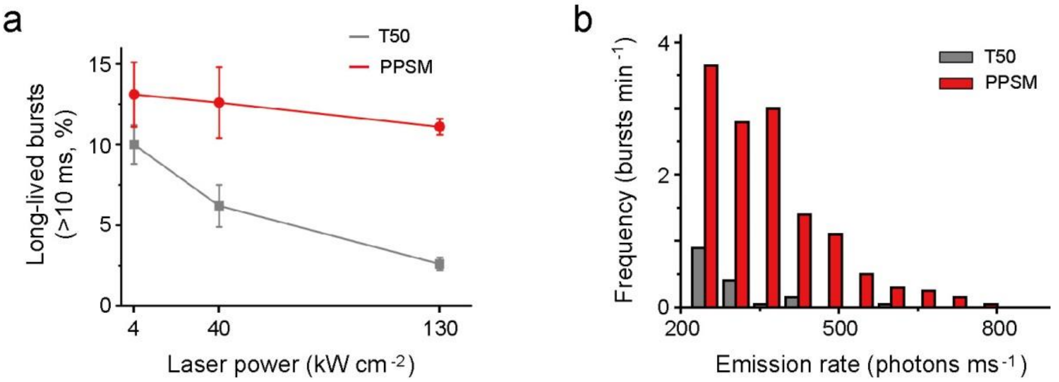

Supplementary Fig. 2. Photo-protection effect of cysteamine – trolox mixture. (a) The fraction of long-lived bursts at various laser intensities. The gray and red lines represent the results for the two buffers, T50 buffer (10 mM Tris-HCl pH 8.0 and 50 mM NaCl) and photo-protection single-molecule buffer (PPSM) (T50 with 10 mM cysteamine and 1 mM trolox), respectively. Error bar represents the standard deviation from three independent measurements. (b) Frequency distribution of the emission rates in long-lived bursts at 130 kW cm^{-2} excitation. When the PPSM buffer was used, approximately 8-fold more bursts were obtained than when the T50 buffer was used.

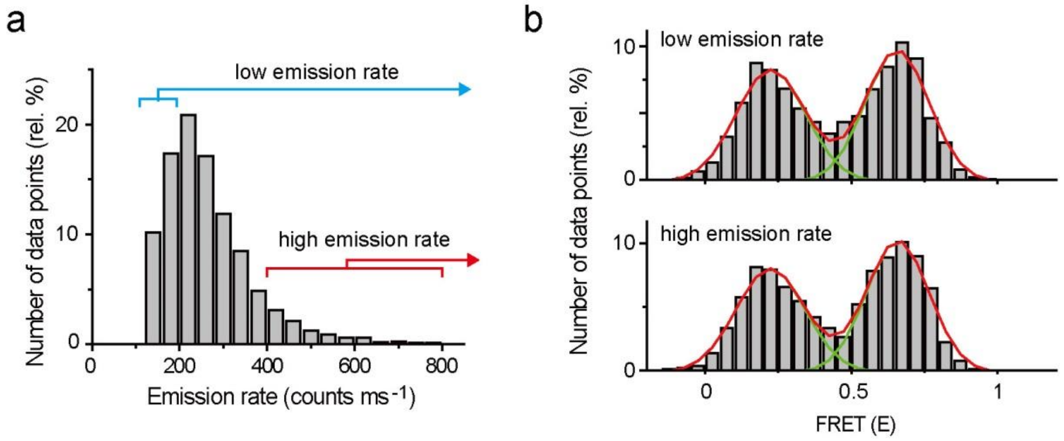

Supplementary Fig. 3. Comparison of the FRET distributions obtained at low and high photon count rates. To confirm that the high photon count rate does not distort the FRET measurement in our conditions, we compared the FRET distributions obtained at low and high photon count rates using a DNA Holliday junction. (a) Distributions of the photon count rate for 1 ms time binning. (b) We selected the time bins that had a photon count rate lower than 200 photons ms^{-1} (low emission rate) or higher than 400 photons ms^{-1} (high emission rate) from (a). The FRET efficiency and the relative population ratios of low and high FRET are nearly identical for both cases: E=0.23 ± 0.03 and 0.65 ± 0.01 for the low emission rate and E=0.23±0.02 and 0.65±0.02 for the high emission rate. The relative populations of low and high FRET were, respectively, 0.48 ± 0.03 and 0.52 ± 0.03 for the low emission rate and 0.46 ± 0.02 and 0.54 ± 0.02 for the high emission rate.

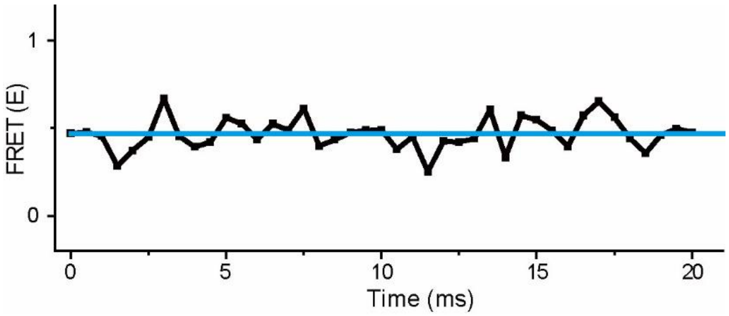

Supplementary Fig. 4. FRET time trace of the DNA Holliday junction in the absence of Mg^{2+}. HMM found only one state without any transition.

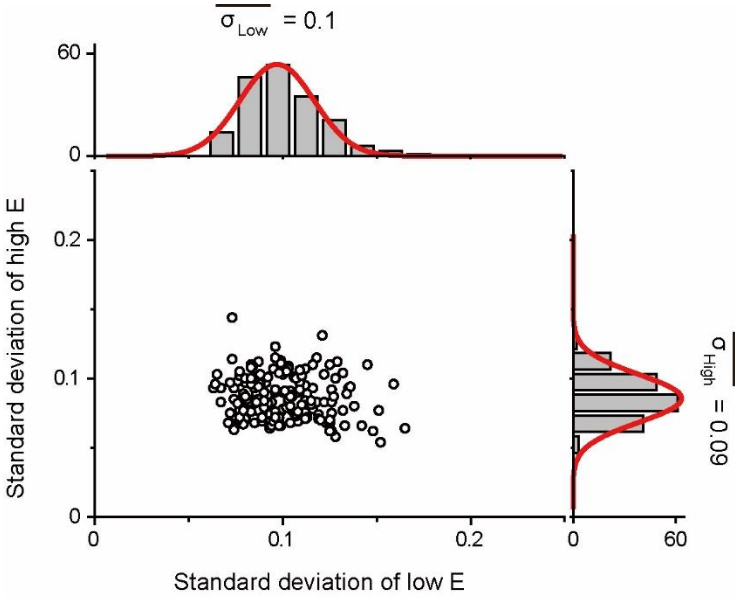

Supplementary Fig. 5. Plot of the standard deviations of FRET efficiency. The standard deviations of FRET efficiency was determined from each time trace of HJ measured at 2 mM Mg^{2+} at room temperature. Each circle in the two-dimensional (2D) scatter plot represents a single time trace: the coordinates of each time trace in the 2D plot denote the standard deviations of the FRET efficiencies of the low- and high-FRET states of the HJ. The 1D distributions of the standard deviations of the low- and high-FRET states are placed on the upper and right sides, respectively.

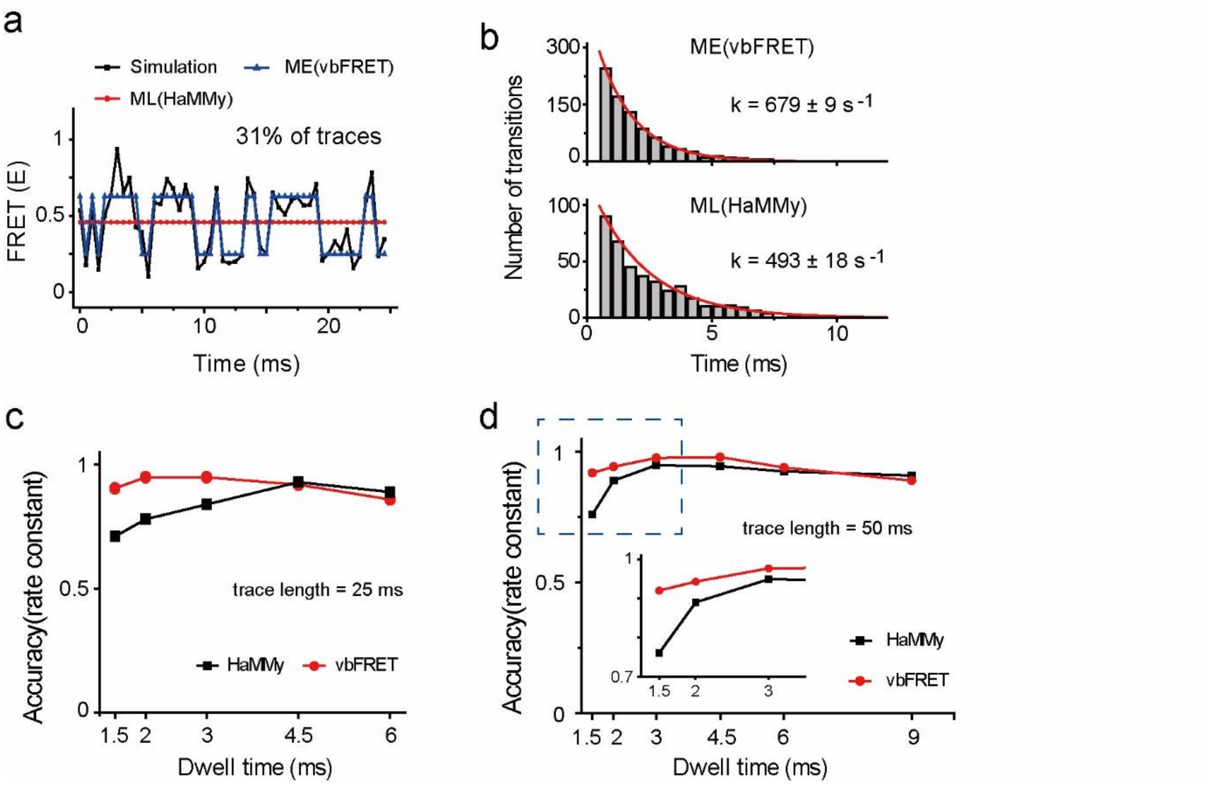

Supplementary Fig. 6. Test of HMM analysis accuracy using synthetic FRET traces. To verify the accuracy of HMM analysis of fast transitions, we applied the HMM methods to simulated synthetic FRET time traces having two-FRET states of E = 0.22 and E = 0.65. The dwell time was varied from 1.44 ms – 6 ms for 25 ms time traces and from 1.44 ms – 9 ms for 50 ms time traces. Synthetic FRET traces were modeled by HaMMy based on the maximum likelihood and by vbFRET based on the maximum evidence. (a) Example synthetic FRET time traces (1.44 ms dwell time). vbFRET correctly found two states for all traces (150 traces), but HaMMy failed to correctly find two states for the 31% of tested traces (47 traces). (b) Dwell time distributions and transition rates from the vbFRET and HaMMy analysis of a 693 s^{-1} transition rate. (c-d) Plots of the accuracy of the two HMM analysis methods depending on the dwell time. The length of the synthetic FRET trace is (c) 25 ms and (d) 50 ms. The actual average length of time traces we used for the analysis in Figure 2 is 26.5 ms.

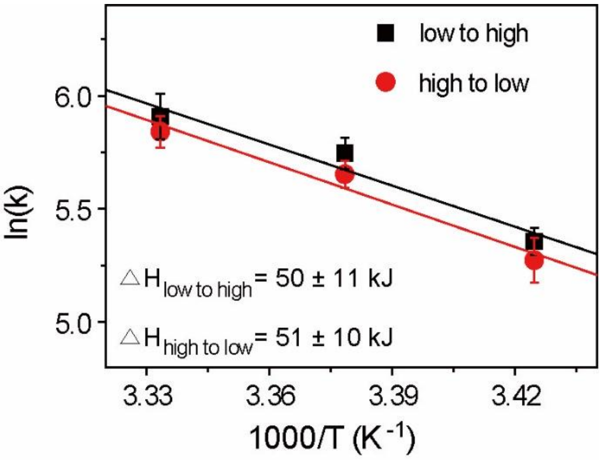

Supplementary Fig. 7. Arrhenius plot of conformer transition of Holliday junction. The Logarithmic values of rate constants plotted as a function of 1/T was fitted with a least-square; temperatures were 19 ℃, 23 ℃, and 27 ℃.

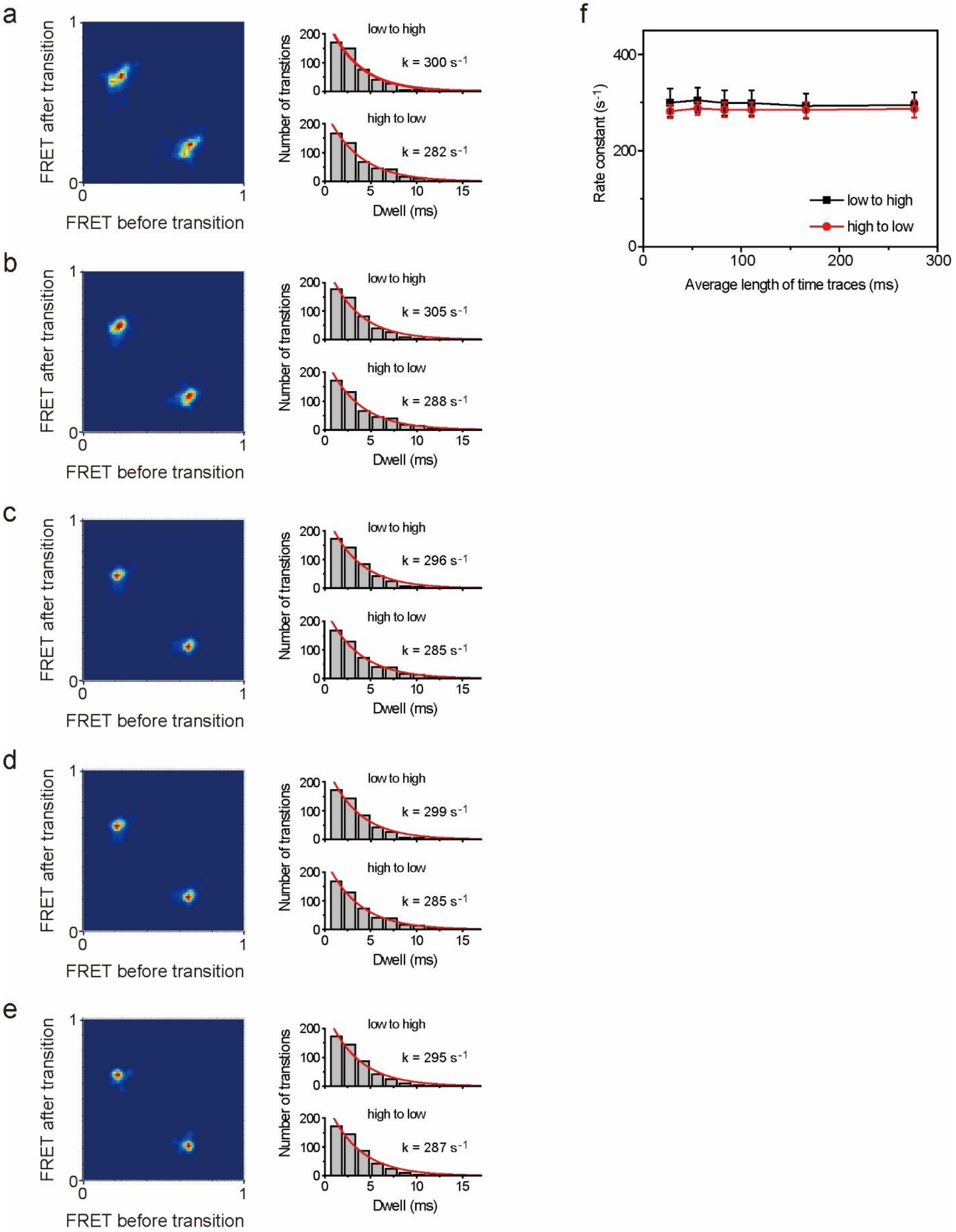
a
b
c
d
e
f
FRET after transition
FRET before transition
Number of transtions
Dwell (ms)
low to high
high to low
k = 300 s-1
k = 282 s-1
k = 305 s-1
k = 288 s-1
k = 296 s-1
k = 285 s-1
k = 299 s-1
k = 285 s-1
k = 295 s-1
k = 287 s-1
Rate constant (s-1)
Average length of time traces (ms)

Supplementary Fig. 8. Stitching effect on transition-density plot and rate constants. (a-e) The transition-density plots (TDPs) and dwell time distributions of HJ at 2 mM $MgCl_2$: (a) no stitching, (b) stitching 2 traces, (c) stitching 3 traces, (d) stitching 4 traces, and (e) stitching 10 traces. (f) The rate constant plot as a function of the average length (0.5 ms binning) of stitched time traces. No significant difference was observed between different numbers of stitching traces. Error bars were obtained from the error of the exponential fit

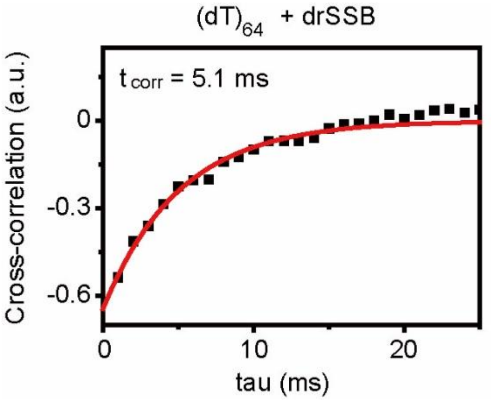

Supplementary Fig. 9. Cross-correlation of $(dT)_{64}$ + *dr*SSB. The correlation time of the FRET fluctuation of $(dT)_{64}$ in the presence of 100 nM *dr*SSB was measured to be 5.1 ms, which is similar to that of $(dT)_{60}$ + *dr*SSB.

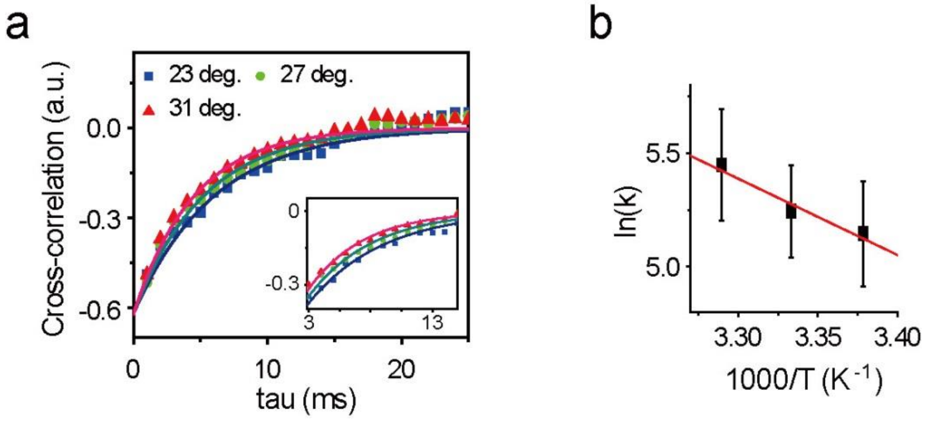

Supplementary Fig. 10. The cross-correlation curves of the FRET fluctuation of $(dT)_{60}$ + *dr*SSB. (a) The cross-correlation curves of the FRET fluctuation of $(dT)_{60}$ + *dr*SSB were obtained at 23 ℃ (blue square), 27 ℃ (green circle), and 31℃ (red triangle), respectively. (b) Arrhenius plot for *dr*SSB diffusion.

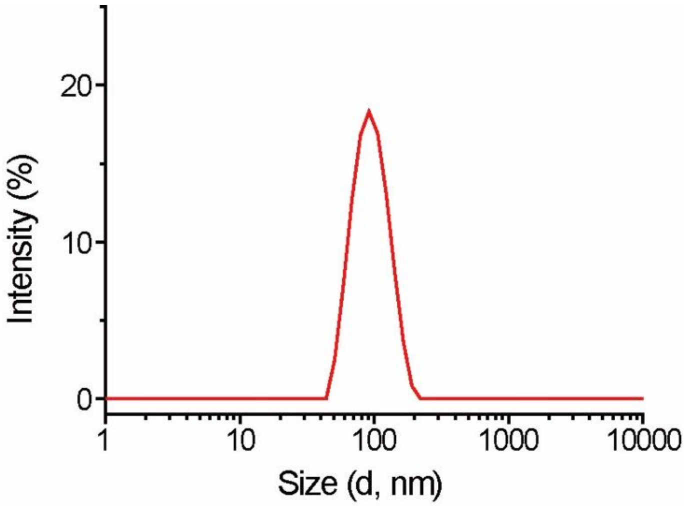

Supplementary Fig. 11. The size distribution of liposomes measured by dynamic light scattering. A dynamic light scattering (DLS) measurement was performed to confirm the size of the liposomes. The DLS measurement was performed using a Zetasizer Nano S instrument (Malvern) with a HeNe laser (633 nm). The scattering was detected at a back-scattering angle of 173° at 25℃. The sample was diluted to a final lipid concentration of 50 μM. The size distribution of the vesicles was analyzed based on the intensity of the vesicles. We observed a monodisperse distribution of vesicles. The average size and polydispersity index of liposomes were 96 nm and 0.073, respectively.

Supplementary Table 1. Parameters for synthetic FRET traces

E values			***Transition probability*** ***(low to high or high to low)***	***Length of time trace***
	State 1	State 2	0.0526 – 0.250 (expected rate : 111 s^{-1} – 693 s^{-1})	25 ms or 50 ms (50 or 100 steps with 0.5 ms binning)
**E (mean)**	0.22	0.65		
**E (std)**	0.1	0.1		

Supplementary Discussion

The probability of multiple binding of HJ to a liposome

When a binding event is an independent process, the probability of binding event has Poisson distribution, $P(x, k) = k^x \exp(-k)/x!$, where x is the number of bound HJ and k is the ratio of HJ and neutravidin, [HJ]/[neutravidin]. Therefore, the probability of multiple binding of HJ to neutravidin is $P(x \geq 2 | x \geq 1) = (P(2) + P(3) + P(4)) / (P(1) + P(2) + P(3) + P(4))$. Because we used HJ: NeutrAvidin=1:100, the probability of multiple binding is ~0.005 (0.5%), which is insignificant. It is to be noted that 0.1% Biotin in liposome gives approximately 50 biotin molecules on outer membrane of a 100-nm sized liposome: here, we didn't intend to put a single biotin to a liposome. We used 1:1 molar ratio of NeutrAvidin: liposome. Because only ~ 1% of NeutrAvidin have HJ, the ratio between HJ-bound NeutrAvidin:liposome is 1:100. Then, again the probability of multiple binding of HJ-bound NeutrAvidin to a liposome is less than 0.5% from Poisson distribution. Considering these two factors, the probability of multiple binding to a liposome is less than 1%. Indeed, when we analyzed time-traces longer than 30 ms with photobleaching steps (22 total), all trace showed single photobleaching step. Thus, the contribution of multiple binding in our measurement is negligible.

Negligible effect of the orientaional fluctuation of dyes

The orientational fluctuation of the probe dyes can induce the fast dynamics we observed. For SSB diffusion on ssDNA, however, the FRET time trace of $(dT)_{60}$ only in Fig. 4b serves as a control measurement to prove that the FRET fluctuations in Fig. 4c and 4d were not caused by the orientational fluctuation of probe dyes. For the dynamics of the DNA Holliday junction, we tested whether the

orientational effect of probe dyes could affect the FRET dynamics by measuring the FRET time traces of the DNA Holliday junction without Mg^{2+}. The stacked conformations of the DNA Holliday junction become unstable in the absence of Mg^{2+}; in this situation, the DNA Holliday junction has only one conformation with an intermediate FRET value (or the transition between two stacked conformations is too fast to resolve using the FRET measurement). Thus, if the FRET fluctuation of the DNA Holliday junction occurred by the orientation fluctuation of the probe dyes, the FRET time trace of the DNA Holliday junction in the absence of Mg^{2+} should show a similar two-state transition. As shown in Supplementary Fig. 4, the FRET time trace of the DNA Holliday junction did not show an apparent two-state transition, and our HMM analysis demonstrated that the trace fit to a single state. Thus, the possible orientational fluctuations of the probe dyes did not cause the FRET fluctuation of the DNA Holliday junction and the SSB movement in our work.

Supplementary Methods

Single-molecule alternating-laser-excitation setup

The microscope setup for alternating-laser excitation (ALEX) has been extensively described elsewhere[1]. In brief, two lasers, a 532-nm solid-state green laser (Cobolt Samba, Cobolt) and a 633-nm HeNe laser (25-LHP-925, Melles-Griot), were used as excitation light sources with 100-μs alternation. The alternation was achieved using acousto-optic modulators (23080-1, Neos Technologies). The two excitation lights were coupled by a dichroic mirror (z532bcm, Chroma) and passed through a single-mode optical fiber (460HP, Thorlabs); they were then collimated and directed into an inverted microscope (IX51, Olympus) and then focused to a point 20 μm from the surface of a coverslip by a water-immersion objective (60×, 1.2 NA, UPLAPO, Olympus) after being reflected by a dichroic mirror (Z532/633RPC, Chroma). The intensities of the 532-nm and 633-nm lasers were typically 1000 μW and 200 μW, respectively, in alternating mode, which were measured before entering the objective. To remove the background intensities from the unfocused region, the fluorescence emissions collected by the objective were focused into a 100-μm pinhole and were then refocused onto silicon avalanche photodiode detectors (SPCM AQR-13, EG&G Perkin Elmer). The emissions of Atto-550 (donor) and Atto-647N (acceptor) were split by a beam splitter (625DCLP, Chroma) and filtered by bandpass filters HQ580/60m and HQ660LP, respectively. The fluorescence time traces were obtained by binning the detected photons into 0.2-ms bins for the 0.5 mM $MgCl_2$ case, 0.3-ms bins for 1 mM $MgCl_2$, 0.5-ms bins for 2 mM and 4 mM $MgCl_2$, and 1-ms bins for 8 mM $MgCl_2$. The data collection and reduction into binned time traces were performed using custom LabVIEW software (National Instruments).

Fluorescence correlation spectroscopy measurements

The fluorescence correlation spectroscopy (FCS) measurements were performed using the same instrumental setup as for ALEX, but a 532-nm continuous laser at a power of 15 μW was used instead of

the alternating-laser conditions. A sample of 2.5 nM oligonucleotide labeled with Atto-647N dye was measured for 4 min at room temperature, and an auto-correlation curve was then obtained using a custom LabVIEW analysis program. For tethering to liposomes, 100 nM NeutrAvidin and 100 nM biotinyl liposomes were added, and the sample was incubated for 10 min prior to the FCS measurement.

Hidden Markov and dwell-time analyses

To identify hidden states and their transitions in the collected time traces of single diffusing molecules, hidden Markov modeling (HMM) was performed using the vbFRET program, which uses a maximum evidence algorithm[2]. We performed an evaluation of the HMM method we used for FRET trace analysis. Among HMM methods, HaMMy and vbFRET are most widely used to analyze FRET traces. The vbFRET uses a maximum evidence (ME), which is more suitable than the maximum likelihood (ML) method used in HaMMy for the analysis of time traces with fast transitions. To verify the accuracy of the HMM methods, we performed HMM analysis using HaMMy and vbFRET on synthetic FRET traces. We generated synthetic FRET traces by simulation using the parameters summarized in Supplementary Table 1; these traces show two-state dynamics with a mean dwell time of 1.44 ms – 9.0 ms between transitions and 0.5 ms temporal resolution. We built analysis code using MATLAB based on the Kevin Murphy Toolbox (http://www.cs.ubc.ca/~murphyk/Software/), which generates discrete FRET time traces that have random transitions between two states. We set two states: $E = 0.22$ and 0.65. Then, we added Gaussian noise to each state with a standard deviation (σ)[2,3]. We used the standard deviation $\sigma = 0.1$ for each state, which was determined from our experimental result (Supplementary Fig. 5). Then, we used two HMM programs to find hidden states in the synthetic FRET traces that had appropriate emission noise. Supplementary Fig. 6 shows a comparison of the performance of the HMM programs that were used to find the hidden states. We found that vbFRET, which is based on ME, correctly found the transitions between the two states for all synthetic FRET traces having 1.44 ms dwell times with a 25 ms

trace length. On the contrary, HaMMy, which is based on ML, correctly found two states for only 69% of all the synthetic FRET traces (Supplementary Fig. 6a). Thus, HaMMy failed to correctly find two state for the 31% of the synthetic FRET traces. Next, we obtained the transition rates from the dwell time distributions obtained from HMM analysis. The theoretical transition rate was 693 s^{-1}, which was calculated from the transition probabilities[4]. The vbFRET (ME) analysis found a value of 624 ± 7 s^{-1}, corresponding to 91 % accuracy. However, the HaMMy (ML) analysis significantly underestimated the transition rate at 493 ± 18 s^{-1}; this value corresponds to a 71% accuracy of the theoretical transition rate, although we excluded the 31% of traces that failed to correctly show two states in the dwell time analysis (Supplementary Fig. 6b). Then, we varied the dwell times from 1.44 ms to 6 ms while fixing the synthetic time-trace length to 25 ms (Supplementary Fig. 6c). vbFRET estimated the transition rates better than HaMMy for fast transitions. When we increased the length of the synthetic time trace to 50 ms, vbFRET was slightly better than HaMMy in estimating the transition rates. Importantly, the accuracy of vbFRET at determining the transition rates was always better than 90%. Thus, vbFRET is appropriate for analyzing the fast dynamic time traces in this work. After HMM, the transition-density plot and dwell-time distributions for each transition were obtained.

Supplementary References

1. Kim JY, *et al.* Solution single-vesicle assay reveals PIP2-mediated sequential actions of synaptotagmin-1 on SNAREs. *EMBO J.* **31**, 2144-2155 (2012).

2. Bronson JE, Fei JY, Hofman JM, Gonzalez RL, Wiggins CH. Learning Rates and States from Biophysical Time Series: A Bayesian Approach to Model Selection and Single-Molecule FRET Data. *Biophys. J.* **97**, 3196-3205 (2009).

3. McKinney S. A., Joo, C. & Ha T. Analysis of single-molecule FRET trajectories using hidden Markov modeling. *Biophys. J.* **91**, 1941-1951 (2006).

4. Andrec M., Levy R. M. & Talaga D. S. Direct determination of kinetic rates from single-molecule photon arrival trajectories using hidden markov models. *J. Phys. Chem. A* **107**, 7454-7464 (2003).
